# Mouse blastomeres acquire ability to divide asymmetrically before compaction

**DOI:** 10.1371/journal.pone.0175032

**Published:** 2017-03-31

**Authors:** Monika Humięcka, Marcin Szpila, Piotr Kłoś, Marek Maleszewski, Katarzyna Szczepańska

**Affiliations:** Department of Embryology, Faculty of Biology, Institute of Zoology, University of Warsaw, Warsaw, Poland; Michigan State University, UNITED STATES

## Abstract

The mouse preimplantation embryo generates the precursors of trophectoderm (TE) and inner cell mass (ICM) during the 8- to 16-cell stage transition, when the apico-basal polarized blastomeres undergo divisions that give rise to cells with different fate. Asymmetric segregation of polar domain at 8–16 cell division generate two cell types, polar cells which adopt an outer position and develop in TE and apolar cells which are allocated to inner position as the precursors of ICM. It is still not know when the blastomeres of 8-cell stage start to be determined to undergo asymmetric division. Here, we analyze the frequency of symmetric and asymmetric divisions of blastomeres isolated from 8-cell stage embryo before and after compaction. Using p-Ezrin as the polarity marker we found that size of blastomeres in 2/16 pairs cannot be used as a criterion for distinguishing symmetric and asymmetric divisions. Our results showed that at early 8-cell stage, before any visible signs of cortical polarity, a subset of blastomeres had been already predestined to divide asymmetrically. We also showed that almost all of 8-cell stage blastomeres isolated from compacted embryo divide asymmetrically, whereas in intact embryos, the frequency of asymmetric divisions is significantly lower. Therefore we conclude that in intact embryo the frequency of symmetric and asymmetric division is regulated by cell-cell interactions.

## Introduction

One of the most crucial events occurring during mammalian preimplantation development is an establishment of two distinct cell populations: the inner cell mass (ICM) and the trophectoderm (TE). They are easily distinguishable in a blastocyst and display different fate, giving rise to the embryo proper and the placenta, respectively. Precursors of the TE and ICM arise when the apico-basally polarized blastomeres of the compacted 8- and 16-cell embryo undergo differentiative divisions, which generate distinct outer and inner cell populations [1–3]. During the compaction the 8-cell stage blastomeres change their morphology: they form adherens junctions and become polarized along the apico-basal axis [4, 5]. Cytoplasmic components, such as microfilaments, endosomes and microtubules accumulate in the apical part of the blastomeres [5–10], while their nuclei reposition basally [11, 12]. At the time when the adhesive lateral junction are formed, blastomeres flatten upon one another and the apical cortical domain enriched in microvilli is established [4]. Many proteins have been shown to participate in the formation of the cortical apical domain, including JAM1, the polarity complex PAR3/PAR/6/aPKC, ezrin and filamentous actin (F-actin) [13–16]. Ezrin is a member of the ERM (Ezrin, Radixin, Moesin) complex, which acts as a cross-linker between F-actin and the plasma membrane. Initially, ezrin is homogeneously distributed at the cell cortex, and becomes restricted to the apical domain during compaction [17, 18]. It has been found that ezrin phosphorylated on treonine T567 (p-Ezrin) interacts with actin filaments and is involved in the formation and stabilization of the apical microvilli domain [19].

During the 8- to 16-cell transition the apical cortical domain disappears but elements of polarity are preserved, allowing blastomeres that inherit the apical region to re-establish polarity and rebuilt the apical domain. During symmetrical division (perpendicular to the apical-basal axis) two polar cells are formed, while asymmetrical division (parallel to the apical-basal axis) give rise to one polar cell and one apolar cell [3]. The polar cells take the outer position, express caudal-like transcription factor 2 (CDX2) and become precursors of the TE [20], whereas apolar cells take the inner position, giving rise to the ICM of the blastocyst [3, 21]. Interestingly, it has been reported recently that the outer or inner position of 16-cell stage blastomeres depends on cellular biomechanical properties, such as cortical tension, mediated by the contractility of actomyosin networks [22–25]. Cortical localization of phospho-myosin light chain II (p-MLCII) observed in inner apolar cells may trigger the engulfment of apolar cells by polar cells [25, 26]. Thus, the cell fate of daughter blastomeres generated after 8- to 16-cell division depends not only on their outer or inner position, but also on the asymmetrical inheritance of the apical domain, which generates blastomeres with different contractilities. This trigger the sorting of cells into outer and inner position, because the less-contractile polar cells tend to engulf more contractile non-polar cells [24].

It is still unknown how the division plane at 8- to 16-cell stage transition is controlled in mouse embryos. Although there is a great variability in the number of inner cells at the 16-cell stage [21, 27], it has been shown that in 8-cell embryo usually at least 5 blastomeres divide asymmetrically [25, 27, 28]. Some authors claim that spindle orientation is random in 8-cell stage blastomeres [6, 29] and others showed that early dividing blastomeres have tendency to divide asymmetrically [30, 31]. It has been also proposed that there is a relationship between the plane of cell division and the position of the nucleus: blastomeres with nuclei located more apically divided symmetrically and blastomeres with basal localization of nuclei prefer to divide asymmetrically [12]. It has been shown that downregulation of proteins, which play the role in establishment of cell polarity, such as PAR3 or aPKC, resulted in higher frequency of asymmetric divisions and increased number of cells contributing to ICM [12, 32]. Moreover, there is a relationship between polarity and expression of CDX2, the TE marker. It has been found that over-expression of CDX2, correlates with increased expression of aPKC and promotes symmetric divisions [33]. Interestingly, blastomeres of compacted 8-cell embryo differ in amount of *Cdx2* mRNA and such heterogeneity may influence their polarity via regulation of aPKC expression, which can affect the orientation of division [26, 33, 34]. It is still not known if the heterogeneity of blastomeres of compacted 8-cell embryo is random [26] or is lineage-related, influenced by specific orientation of cleavage divisions during the 2→4 cell transition [35, 36, 37]. Recently it has been shown that at the 4-cell stage the gene expression is heterogeneous between individual blastomeres and this may initiate cell-fate decisions, reflected through definite division patterns [38]

It is known that single blastomeres isolated from a compacted 8-cell embryo maintain the ability to divide *in vitro* asymmetrically, and that cell interactions do not influence the orientation of the division plane [3, 6]. Some authors claim that number of asymmetric divisions in isolated polar blastomeres varies between embryos [26] and that cleavage plane during the 8–16 cell division is not predetermined. However, our previous studies suggested that the division plane in blastomeres of a compacted 8-cell embryo is already determined and cannot be affected by cell interactions [39]. We found that the total number of CDX2-positive cells in embryo fragments developed after 2 rounds of divisions from 8 single 8-cell stage blastomeres isolated from the compacted embryo is similar to that observed after 2 rounds of divisions in the intact embryo.

In the present study we wished to explore when in the 8-cell stage blastomeres the division plane becomes determined, and how it depends on intercellular interactions. To this end we followed division pattern of single blastomeres isolated from 8-cell embryos before and after compaction. Using p-Ezrin as a polarity marker we were able to identify asymmetrical and symmetrical divisions. We found that at early 8-cell stage, before any visible signs of cortical polarity, a subset of blastomeres had been already predestined to divide asymmetrically. This indicates that a mechanism controlling the division plane, and therefore cytoplasmic polarization of the blastomeres exist before compaction and formation of the apical cortical domain. Moreover our results revealed that, while most of the blastomeres of uncompacted embryo divide symmetrically, almost all of 8-cell stage blastomeres of compacted embryo are predestined to divide asymmetrically. We also showed a reduction in a number of asymmetric divisions in the intact embryo compare with isolated blastomeres, probably as the result of regulation frequency of symmetric versus asymmetric divisions by cell-cell contacts in the embryo.

## Materials and methods

The study was approved by the Local Ethic Committee No.1, Warsaw, Poland. (Permit number 679/2015)

All chemicals were purchased from Sigma-Aldrich (Poznan, Poland), unless stated otherwise.

### Embryos collection

Embryos were obtained from F1(C57Bl/6Tar x CBA/Tar) 8–12 week-old female mice, which were induced to ovulate by injection of 10 IU of PMSG (Intervet, Poland) followed 48 hr later by 10 IU of hCG (Intervet, Poland), and mated with F1(C57Bl/6Tar x CBA/Tar) male mice. Female mice were sacrificed by cervical dislocation and males remained after the experiment in a breeding colony in our animal house. Embryos at the 4-cell stage and compacted 8-cell stage embryos were obtained from the oviducts and uteri 60 and 67 hours after hCG injection, respectively. Control 16-cell stage embryos were isolated 74 hours after hCG injection. Embryos were cultured *in vitro* at 37.5°C in 5% CO_2_ in air, in M2 medium (medium M16 buffered with HEPES, [40]) supplemented with 4mg/ml BSA or in KSOM ([41], Specialty Media, USA) under mineral oil.

### Embryo disaggregation and culture of individual blastomeres

The individual blastomeres (named 1/8 blastomeres) were obtained from uncompacted 8-cell embryos (immediately after the transition from 4-cell stage) or compacted 8-cell embryos. In order to separate blastomeres both groups of embryos were freed of zonae pellucidae using acid Tyrode’s solution (pH 2.5, [42]) and incubated for 1 hr in M2 medium without Ca^2+^ and Mg^2+^, supplemented with EGTA (0.2 mg/ml). Single blastomeres were obtained either by gentle pipetting or separated mechanically with a glass needle. After the dissociation, each 1/8 blastomere was transferred to a separate well of a Terasaki plate (Sarstedt, Poland) and cultured for 5 to 8 hours in KSOM under mineral oil in 37,5°C in 5% CO2 in air, until it divided to two 16-cell stage (2/16) blastomeres. Every 30 min the blastomeres were checked visually for signs of division and the type of division for each 1/8 blastomere was recorded. The divisions were described as equal if both blastomeres in 2/16 pair were of equal size, or unequal—if one of the blastomeres in 2/16 pair was bigger than another. Each pair of 2/16 blastomeres was cultured until compaction was observed and after that they were fixed for p-Ezrin immunodetection.

The control (intact) 2/16 pair of blastomeres was obtain from 16-cell stage embryos, which were disaggregated by gentle pipetting after 1 hr of incubation in M2 medium without Ca^2+^ and Mg^2+^, supplemented with EGTA (0.2 mg/ml) as has been described above.

Some of 2/16 blastomere pairs were cultured for about 24-28hrs i.e. until the next division (until control intact 8-cell stage embryos started cavitation at approximately 32-cell stage). During that time the 2/16 blastomeres divided and formed an embryo fragment, which corresponded to one eighth of the 32-cell stage (4/32 quartet of blastomeres). The control embryos and experimental embryo fragments were fixed for immunodetection of CDX2.

### Immunodetection of p-Ezrin and CDX2 protein

Control 2/16 pairs of blastomeres and experimental embryo fragments (2/16 pairs and 4/32 quartets) were fixed in 4% PFA for 40 min at room temperature, and then permeabilized with 0.3% Triton X-100 for 15 min. The nonspecific antibody binding was blocked by incubation in 3% BSA (for CDX2 staining) or in 10% FBS (for p-Ezrin staining), overnight, at 4°C. Embryos and embryo fragments were then incubated with primary antibodies (mouse monoclonal anti-CDX2 antibody (1:50, BioGenex, USA) or rabbit monoclonal antibody anti-p-ERM (phospho-Ezrin(Thr567)/Radixin(Thr564)/Moesin(Thr558), 1:400, Cell Signaling Technology, 3141) at 4°C, overnight, and then treated with secondary antibodies (TRITC-conjugated goat anti-mouse antibody (1:200, Jackson ImmunoResearch) or goat anti-rabbit AlexaFluor 633 (1:200, Molecular Probes)) for 1 hour at room temperature. Additionally, chromatin was stained by incubation with DRAQ5 (10 μM; Biostatus, Leicestershire, UK) or chromomycin (0.01 mg/ml) for 10 min at 37°C, and F-actin by incubation with FITC-conjugated phalloidin (1:1000) for 20 min at room temperature. The embryos and embryos fragments were analyzed using a Zeiss 510 confocal laser microscope.

### Statistical analysis

Statistical analyzes were performed either using chi-squared test.

## Results

### A subset of blastomeres of uncompacted embryo is already predetermined to asymmetric divisions

**Fig 1:**
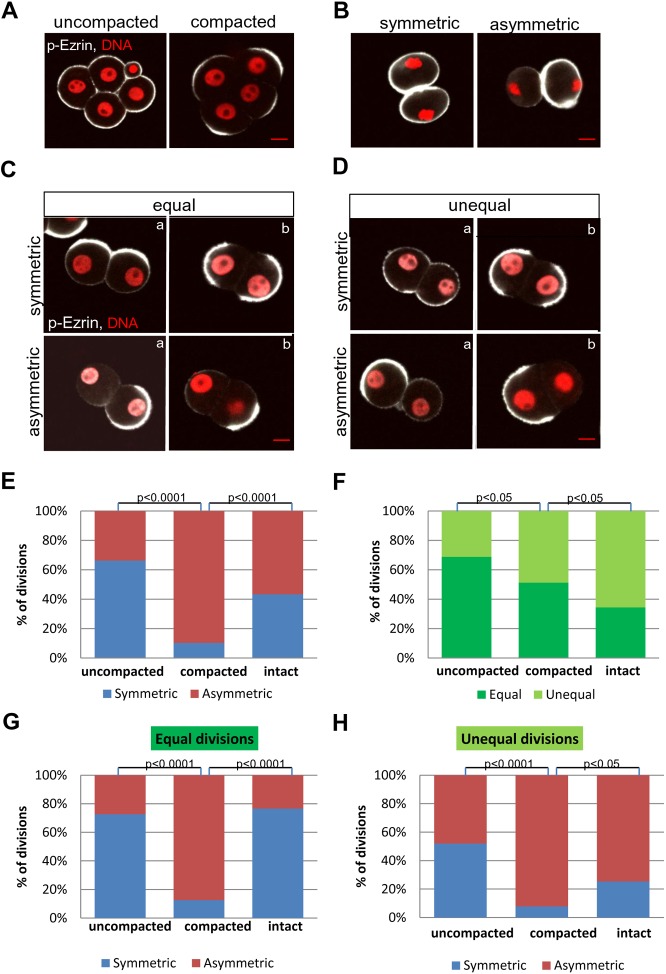
Pattern of divisions of blastomeres isolated from uncompacted and compacted embryos and intact embryos. **(A)** p-Ezrin uniformly distributed in their cortex of cells of uncompacted eight-cell embryo and restricted to the apical domain of cells in compacted embryo. (**B)** Symmetric and asymmetric inheritance of p-Ezrin during divisions of 1/8 blastomeres isolated from uncompacted and compacted embryos. (**C)** symmetric and asymmetric divisions in group of equally sized blastomeres in 2/16 pair. a) p-Ezrin uniformly distributed in cortex of blastomeres before compaction. b) p-Ezrin restricted to the apical domain of blastomeres after compaction. (**D)** symmetric and asymmetric divisions in group of unequally sized blastomeres in 2/16 pair. a) p-Ezrin uniformly distributed in cortex of blastomeres before compaction; b) p-Ezrin restricted to the apical domain of blastomeres after compaction. Scale bar 10μm. **(E,F,G,H)** Analysis of symmetric/asymmetric and equal/unequal divisions of 1/8 blastomeres isolated from uncompacted (n = 80), compacted 8-cell stage embryos (n = 78) and blastomeres of intact embryos (n = 90). The distribution of p-Ezrin was estimated by immunofluorescence after fixation of 2/16 pairs of blastomeres.

It has been shown previously that individual blastomeres isolated from compacted 8-cell stage embryos remain predetermined in respect to the plane of the 8- to 16-cell division [26, 39]. In order to examine when this determination of the prospective division plane occurs, we compared division patterns in blastomeres isolated from early 8-cell embryos before compaction (at 60 hp-hCG), and late 8-cell embryos after compaction (at 67 hp-hCG). To examine the status of polarization of blastomeres at the time of isolation, we used p-Ezrin as a polarity marker and analyzed its localization in blastomeres of intact early and late 8-cell embryos. We observed that the blastomeres from the uncompacted embryos have not formed the apical domain yet since p-Ezrin was uniformly distributed in their cortex, whereas after the compaction p-Ezrin was clearly restricted to the apical domain of blastomeres (Fig 1A).

Next, the individual 1/8 blastomeres isolated from uncompacted and compacted embryos were cultured *in vitro* until 70–72 h post hCG (i.e. for 7 or 2 hrs, respectively) when most of them divided to 2/16 blastomere pairs. The divisions of 1/8 blastomeres were classified by size of sister blastomeres in the 2/16 pair: as equal, when both cells had the same size, or as unequal, when the cells differed in size.

To examine the relationship between the size of blastomeres and their polarity, we analyzed the localization of p-Ezrin in 2/16 blastomere pairs of equal and unequal size. We classified division as symmetric when p-Ezrin was present in both sister 2/16 blastomeres, and as asymmetric division when p-Ezrin was inherited only by one of the blastomeres. We found that 1/8 blastomeres isolated from both uncompacted and compacted embryos could divide symmetrically as well as asymmetrically. Initially, just after division, p-Ezrin was equally distributed in the cortex (Fig 1B) and during the next 30–60 minutes, when pair of 2/16 blastomeres underwent compaction, p-Ezrin was located in apical domain of cells (Fig 1C and 1D). Interestingly, we observed that both symmetric and asymmetric divisions of 1/8 blastomeres could result in equal or unequal size daughter blastomeres. It was characteristic, that in case of asymmetric and unequal division, the polar cell, inheriting p-Ezrin was always bigger than the apolar one (Fig 1D).

We analyzed eighty 2/16 blastomere pairs derived from 1/8 blastomeres of uncompacted 8-cell embryos and seventy eight pairs of 2/16 blastomeres obtained from 1/8 blastomeres of compacted 8-cell embryos. This analysis revealed that although most of the 1/8 blastomeres from uncompacted embryos divided symmetrically (66.3%, n = 53/80), unexpectedly up to 33% of them divided asymmetrically (Fig 1E). In contrast, the blastomeres from compacted 8-cell embryo underwent mostly asymmetric divisions (89.7%, n = 70/78, p<0.0001). We found that 1/8 blastomeres derived from uncompacted 8-cell embryos underwent equal divisions more often (68.8%, n = 55/80) than unequal divisions (31.2%, n = 25/80, Fig 1F). In contrast, blastomeres from compacted 8-cell embryo divided equally and unequally with similar frequencies (51.3%, n = 40/78 vs 48.7%, n = 38/78). Observed differences in proportion of equal and unequal divisions between blastomeres of uncompacted and compacted was statistically significant (p<0.05). Moreover, when we compared equal/unequal divisions with symmetric/asymmetric ones in blastomeres of uncompacted embryos, we noticed that although 72.7% of 2/16 pairs (n = 40/55) with equally sized sister blastomeres arose from symmetric divisions, still significant proportion of 2/16 pairs composed of blastomeres of the same size (27.2%, 15/55) inherited p-Ezrin domain asymmetrically. We also found that 2/16 pairs with unequally sized sister blastomeres could result from both symmetric and asymmetric divisions in similar proportion (52% and 48%, n = 13/25 and 12/25 respectively, Fig 1H). In contrast, in the case of compacted embryos, both equally and unequally sized 2/16 pairs originated predominantly from asymmetric divisions (87.5% and 92.1%, n = 35/40 and 35/38, respectively). Observed differences in proportion of asymmetric/symmetric divisions among equal/unequal between blastomeres of uncompacted and compacted was statistically significant (p<0.0001).

Taken together, this analysis shows that isolated blastomeres derived from uncompacted 8-cell embryos most frequently divide symmetrically, while blastomeres isolated from compacted embryos are biased towards asymmetric divisions. However, we also found that a subset of blastomeres have already acquired the ability to undergo asymmetric divisions before the compaction. Importantly, we also proved that asymmetrical distribution of p-Ezrin does not necessarily correlate with the difference in the size between sister blastomeres, and therefore the presence of differentially sized blastomeres cannot be used as a marker for asymmetry of division as defined by inheritance of cytoplasmic and/or membrane components.

### Blastomeres of intact 8-cell embryo less often undergo asymmetric divisions than isolated blastomeres

To estimate the frequency of symmetric and asymmetric divisions of blastomeres of intact 8-cell stage embryo, we analysed ninety 2/16 pairs of blastomeres obtained after dissociation of 16-cell stage embryos. We found that 56.7% (n = 51/90) of 2/16 pair of blastomeres in intact embryos were the result of asymmetric divisions of 1/8 blastomeres, which is significantly less than 89.7% of frequency of asymmetric divisions of 1/8 blastomeres isolated from compacted 8-cell stage embryo (Fig 1E). Moreover we found that although blastomeres in intact embryo underwent unequal divisions more often than isolated 1/8 blastomeres (64.6% vs 48.7%, p<0.05, Fig 1F), there were more frequently symmetric in intact embryos compared to isolated blastomeres from compacted embryo in both equal (74.4% vs 12.5%, p<0.0001, Fig 1G) and unequal divisions (25.4% vs 7.9%, p<0.05, Fig 1H).

### Blastomeres from uncompacted and compacted embryos generate different patterns of CDX2 expression after two rounds of division

**Fig 2:**
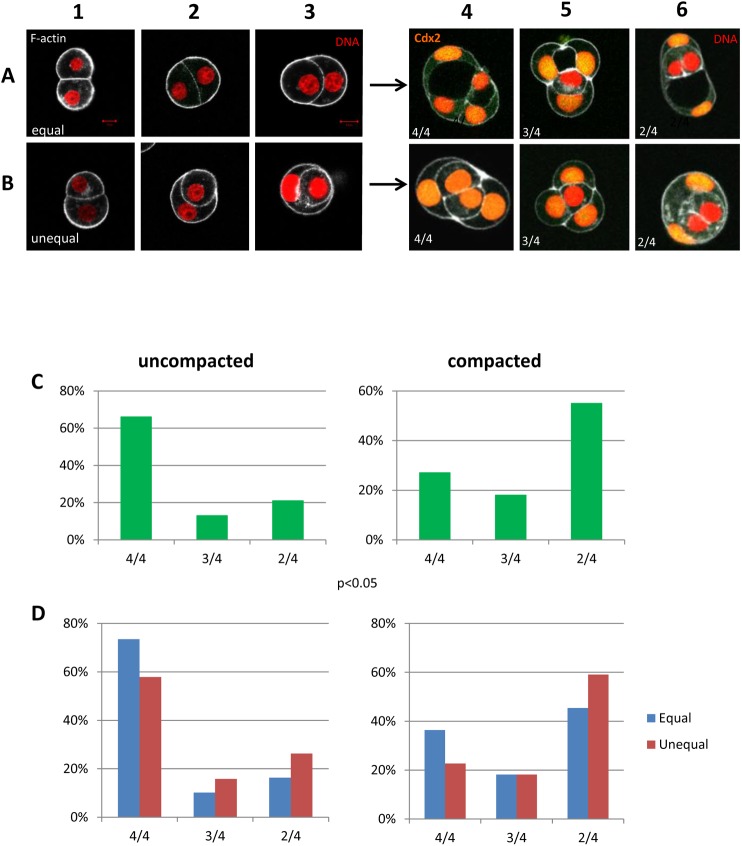
Expression of CDX2 in embryo fragments after equal and unequal divisions of blastomeres isolated from uncompacted and compacted embryos. **(A)** Equal division of 1/8 blastomeres—1) 2/16 pairs of blastomeres before compaction. 2,3) 2/16 pairs of blastomeres after compaction. The borders of cells are visualized by actin staining with phalloidin (white). 4,5,6) Embryo fragments composed from 4 cells generated after the next round of division with different number of CDX2-positive cells—4/4, 3/4, 2/4 (number of CDX2-positive cells/number of cells). All images are merged single optical images of chromatin (red) and CDX2 (yellow) staining. Scale bar 10μm. (**B)** Unequal division of 1/8 blastomeres—1) 2/16 pairs of blastomeres before compaction, 2,3) 2/16 pairs of blastomeres after compaction. The borders of cells are visualized by actin staining with phalloidin (white). 4,5,6) Embryo fragments composed from 4 cells generated after the next round of division with different number of CDX2-positive cells (4/4, 3/4, 2/4). All images are merged single optical images of chromatin (red) and CDX2 (yellow) staining. Scale bar 10μm. (**C)** Frequency of embryo fragments with different number of CDX2-positive cells (4/4, 3/4, 2/4) developed from 1/8 blastomeres of uncompacted and uncompacted embryos. (**D)** Frequency of embryo fragments with different number of CDX2-positive cells developed from 1/8 blastomeres of uncompacted and uncompacted embryos after equal and unequal divisions.

In order to estimate how the cell-cell interactions in the 2/16 pair of blastomeres determine the number of polar cells after the next divisions, we cultured them until both cells underwent division. Because the inheritance of the polar domain determines CDX2 expression in the descendant cells [2,34], we used CDX2 as the marker of polar cells within the 4/32 fragments of embryo. We analyzed separately the 2/16 pair formed after equal and unequal divisions of 1/8 blastomeres of uncompacted and compacted embryos (Fig 2A and 2B). We observed that blastomeres in 2/16 pair underwent compaction during first 30–60 minutes after division, which is manifested by the increase of the surface of adhesion between cells (Fig 2A and 2B1). During the next 3 hours the interactions between cells changed depending on the size of sister 2/16 blastomeres. Equally sized blastomeres enhanced adhesion between each other and formed a spherical shape with flat or slightly curved contact surface (Fig 2A2 and 2A3). If cells in the 2/16 pair differed in size, soon after compaction the smaller blastomere was partially (Fig 2B2) or completely enveloped by the bigger one (Fig 2B3).

Both equal and unequal 2/16 blastomeres pairs after the next 7–8 hours underwent division, forming 4-cell fragments (4/32 blastomere quartets) with different cell arrangements and diverse pattern of CDX2 expression: 4/4 –where CDX2 was expressed in all of 4 cells, 3/4—CDX2 in 3 of 4 cells, and 2/4 –CDX2 in 2 of 4 cells (Fig 2A and 2B4–2B6). We found that in all 4-cell fragments originating from the equally sized 2/16 pairs, CDX2-negative cells had outer position or were partially surrounded by CDX2-positive cells, which formed cavity between each other. (Fig 2A4–2A6). In contrast, unequal 2/16 pairs generated fragments with CDX2-negative cells always enveloped by outer CDX2-positive ones (Fig 2B4–2B6). We found that blastomeres isolated from uncompacted embryos most frequently (66%, n = 58/87) formed 4/4 fragments, while blastomeres from compacted embryos preferentially (55%, n = 36/66) generated 2/4 fragments (Fig 2C). Moreover, our results showed that in case of blastomeres originating from uncompacted embryos the 4/4 arrangement was predominant in fragments derived from both equally and unequally sized 2/16 pairs (73.5% and 57.9% of fragments, respectively, Fig 2D). Analogically, in case of blastomeres from compacted embryos, both equal and unequal 2/16 pairs produced preferentially 2/4 fragments (45.4% and 59.1% of fragments, respectively, Fig 2D).

**Fig 3:**
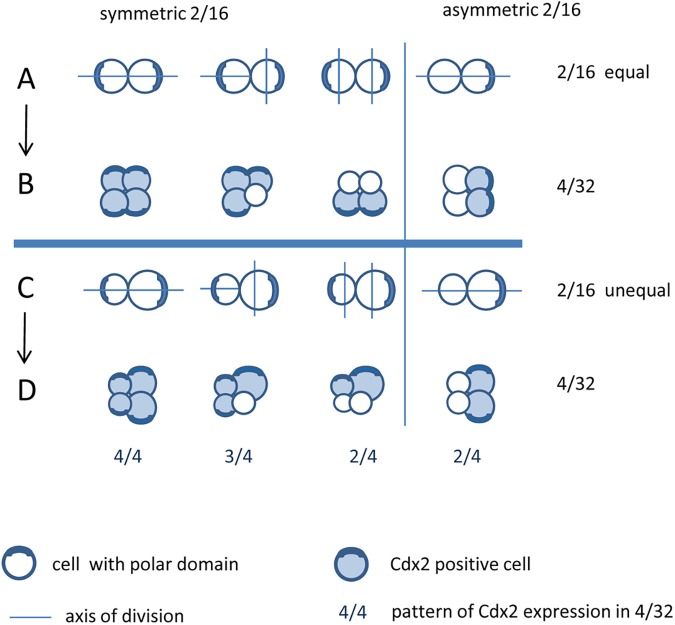
The model of cell division of 2/16 pair of blastomeres developed in symmetric/asymmetric and equal/unequal divisions of 1/8 blastomeres.

To explain various CDX2 expression patterns in 4-cell embryo fragments obtained from blastomeres from uncompacted and compacted embryos, we created the model, which takes into account all possible consecutive cell divisions (equal and unequal, symmetric and asymmetric) of 2/16 pair of blastomeres (Fig 3). The subsequent divisions of 1/16 blastomeres in 2/16 doublet (Fig 3A and 3C), give rise to formation all of obtained patterns of CDX2 expression in 4/32 quartets (4/4, 3/4, 2/4, Fig 3B and 3D). The asymmetric 2/16 pairs of blastomeres (both equal and unequal), mostly originated from blastomeres of compacted embryo (Fig 1F, 1G and 1H) during the next round of division produced preferentially fragment with 2/4 of CDX2 expression (Fig 2C and 2D). So, we can conclude that the polar cell in 2/16 pair of blastomeres always undergoes a symmetric division as it is shown in Fig 3. The asymmetric division of the polar cell would result in 1/4 pattern of CDX2 expression, which was never observed in our experiments. In contrast, 1/8 blastomeres isolated from early, uncompacted 8-cell embryos divided mostly symmetrically (Fig 1F), giving equally or unequally sized 2/16 blastomeres (Fig 3). As they usually develop into fragments displaying the 4/4 pattern of CDX2 expression (Fig 2C), it seems likely that the two polar cells in 2/16 pair of blastomeres divided mostly symmetrically (Fig 3).

**Fig 4:**
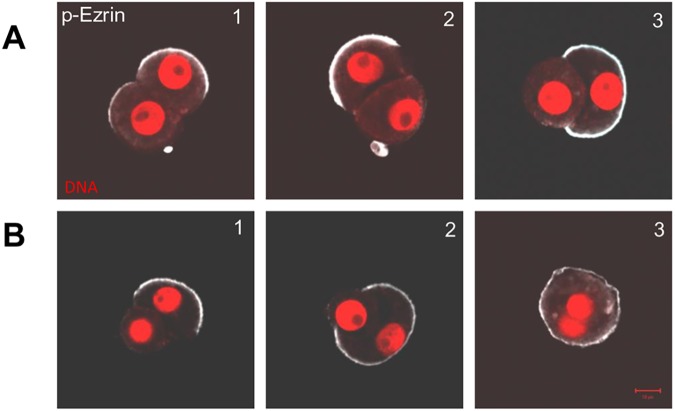
Cell-cell interactions in the 2/16 pair of blastomeres. **(A) 1.** Symmetric equal division–p-Ezrin in both blastomeres. **2**. Asymmetric equal division–p-Ezrin in one of the blastomere. **3**. Asymmetric unequal division–p-Ezrin in the bigger blastomere. (**B)** Asymmetric unequal division. **1,2,3** Sequence of enveloping the small apolar blastomere by the polar bigger one. Scale bar 10μm.

Moreover, we found that cell-cell interactions correlate with the CDX2 expression, which was the most visible in 2/4 fragments. If 1/8 blastomeres divided unequally and asymmetrically (Fig 2B3, Fig 3), the small cell had been surrounded by the bigger one before both of them underwent the next division, giving rise to two big outer CDX2 positive cells and two small inner CDX2 negative cells (Fig 2B6, Fig 3, Fig 4B).

The 2/4 pattern of CDX2 expression with similar arrangement of 4/32 cells could also originate from symmetric, equal division of 1/8 blastomere (Fig 3, Fig 4A1), but in this case the next round of division seemed to be asymmetric and unequal. Interestingly, we observed that difference in size in 2/16 pair of blastomeres did not always determine the ability to envelop and internalize one cell by another. In the case of symmetric and unequal 2/16 blastomeres, after the next round of symmetric divisions all cells in 4/4 fragments were polar and outer, and expressed CDX2, although they were different in size (Fig 3D). In contrast, if all cells in the 4/32 quartet were equal in size but originated from asymmetric division of 1/8 blastomere (Fig 4A2), the two CDX2 positive cells partially surrounded the CDX2 negative cells and sometimes such fragment formed a cavity (Fig 2A3–2A6, Fig 3).

In summary, we found that the cell-cell interactions in cleaving mouse blastomeres do not depend on blastomere size, but on the presence of polar domain, which is inherited during the subsequent divisions by one (asymmetric division) or two cells (symmetric division).

The pattern of CDX2 expression in 4/32 fragments of embryos (4/4, 3/4 or 2/4) depends on the symmetric or asymmetric inheritance of polar domain during the 2/16 to 4/32 division. The 1/8 blastomeres isolated from compacted 8-cell stage embryos underwent mostly asymmetric (equal or unequal) divisions, giving mostly 2/4 pattern of CDX2 expression. In contrast, 1/8 blastomeres isolated from early, uncompacted 8-cell embryos divided mostly symmetrically (equally or unequally) and usually develop into fragments displaying the 4/4 pattern of CDX2 expression.

## Discussion

It is known that apico-basal polarization of 8-cell stage blastomeres influences the fate of their descendents arising from symmetric and asymmetric divisions [43]. Blastomeres, which inherit the cortical apical domain, represented by microvilli/p-ERM/F-actin, become polarized and take outer position in 16-cell embryo, whereas cells which do not inherit the polar domain are apolar and localize inside the embryo [43]. The former group of cells starts expressing CDX2 transcription factor and gives rise to TE, while the latter group, CDX2-negative, gives rise to the ICM of the prospective blastocyst [1, 44–46]. Our previous results indicated that the pattern of division (asymmetric or symmetric) in blastomeres is already established in compacted 8-cell embryo, because after the next two divisions of single blastomeres isolated from the embryo, the number of CDX2-positive cells (i.e. the number of polar cells) is similar to that in the intact embryo [39]. This suggested, that single blastomeres isolated from compacted embryos maintained the predetermined division plane and memory of how to divide–symmetrically or asymmetrically.

In the present study we addressed a question whether apico-basal polarization of the 8-cell stage blastomeres is a prerequisite for determination of the division plane. Our rationale was that blastomeres of early uncompacted 8-cell embryos are still apolar and after isolation from the embryo they will not polarize during several hours of in vitro culture. Therefore we hypothesized that all of them will divide symmetrically forming 2/16 pair of blastomeres, which would gradually begin to polarize, giving two polar cells. On the other hand, single blastomeres from 8-cell compacted embryo were already polarized and were supposed to divide both symmetrically and asymmetrically, as shown previously by other authors [25, 26]. We distinguished polar and apolar cell in the pair of 2/16 blastomeres by localization of p-Ezrin localized in the apical part of polarized cells. The p-Ezrin is a reliable polarity marker in mouse blastomeres because it is directly associated with the pole of microvilli [25].

However, unexpectedly, our analysis of the localization of p-Ezrin in pairs 2/16 showed that up to 34% of blastomeres isolated from early uncompacted 8-cell stage embryo divided asymmetrically, although they had no signs of cortical polarity before the division. It is difficult to explain why blastomeres from uncompacted early 8-cell embryo undergo asymmetric divisions (we checked that they were not cortically polarized just after isolation from embryo and after 7 hours of culture *in vitro*) (Szczepańska, not shown). We speculate that a mechanism controlling the division plane and therefore probably cytoplasmic polarization of blastomeres exist before compaction and formation of the apical domain. It has been suggested that heterogeneity of blastomeres at 8-cell stage is nonrandom and relates to different orientation of early cleavage division, thus inheritance of different part of zygote [35, 47]. Other authors found that blastomeres at 8-cell early stage are different in kinetics of pluripotent factor Oct4, before any morphological differences are visible [48].

Our results revealed also that almost all 1/8 blastomeres isolated from compacted embryos underwent asymmetric divisions. This value is much higher than frequency of asymmetric divisions observed in the intact 8-cell embryo (usually only about 60% (5 of 8) blastomeres divides asymmetrically [3, 25, 27, 28]. Our analysis of 2/16 pair of blastomeres isolated from intact 16-cell stage embryo also showed that only 56% divisions during 8–16 transition were asymmetric. Therefore, it seems that cell-cell contacts in the intact embryo, or, more generally, embryo geometry, regulate the proportion of symmetric and asymmetric divisions, reducing the number of the latter [49–51].

We also observed that polar and apolar blastomeres indeed differ in their biomechanical properties, what is reflected by the patterns of interactions of sister cells in 2/16 and 4/32 fragments. We observed that in 2/16 blastomere pairs the polar cell enveloped the apolar cell, whereas in case of two polar cells such interaction was never noticed. It has been shown that cortical contractility of polar and apolar cells differs, at least partially due to the fact that apolar cells accumulate more phospho-myosin light chain II (p-MLCII) in their cortex than polar blastomeres [22, 24, 25]. Such an uneven p-MLCII distribution enables engulfing of the apolar cell by the polar one. Interactions analogous to these recorded for isolated blastomeres can be also seen in the intact embryo, where apolar cells are internalized by the surrounding polar cells [22–25, 52, 53].

Our results revealed also that in both symmetric and asymmetric divisions of 1/8 blastomere the daughter cells could be equal or unequal in size. Interesting, we found that also in intact embryo cells of different size could be generated in both symmetric and asymmetric divisions. Thus, size of blastomeres in 2/16 pairs cannot be used as a criterion for distinguishing symmetric and asymmetric divisions, as was suggested previously [3, 26]. We also found out how the cell-cell interaction of equally and unequally sized blastomeres correlates with the CDX2 expression after the next round of divisions, when 4/32 quartet of cells was formed. Our previous work [39] showed, that blastomeres isolated from compacted 8-cell embryo developed mostly into the 2/4 fragments, where two bigger CDX2 positive cells enveloped two smaller cells CDX2 negative cells. Here we showed that this pattern of CDX2 expression resulted from unequal asymmetric division of 1/8 blastomere. We also found that 2/4 fragment could originate from equal asymmetric division of 1/8 blastomere and, with a lower frequency, from equal symmetric division. We could distinguish these two variants by the arrangement of cells in the fragments: the CDX2 positive cells engulfed partially or completely CDX2 negative cells. Moreover, using the blastomeres from both uncompacted and compacted embryo we were able to observe how the increasing tendency for asymmetric division is reflected in a change of CDX2 expression pattern in 4/32 fragments from 4/4 to 2/4. We also showed that the cell-cell interactions, such as engulfing small cell by the bigger one, depend not on size of the cells, but on presence of the polar domain. In the 4/4 fragments derived from unequal symmetric divisions, all four cells maintained outer characteristics despite the difference in size, because they all were polar.

Taken together, our studies on blastomeres isolated from 8-cell embryo suggest that blastomeres begin to polarize asynchronously long before compaction and before the formation of cortical apical domain. We think that the ability to divide asymmetrically is developing gradually in individual blastomeres before and during the compaction, as vast majority of blastomeres isolated from compacted embryos are biased towards the asymmetric division. It seems likely that differences in blastomere ability to undergo an asymmetric division result from the asynchrony in cleavage divisions at 4- to 8-cell stage transition. The 8-cell stage blastomeres that were formed first, stayed longer in contact with other cells and thus might acquire properties predestining them to the asymmetric divisions.

Our analysis revealed that the frequency of asymmetric divisions observed in isolated single 1/8 compacted blastomeres is much higher than an average frequency recorded for whole intact embryos, which indicates that in the intact embryo cell-cell contacts and mechanical constraints reduce the number of asymmetric divisions to the most optimal. Taken together we showed that although the potential to divide asymmetrically is developed in each blastomere independently during compaction, the final number of asymmetric divisions, i.e. the realization of this potential, is controlled within the embryo by cell-cell interactions.
